# Sulfasalazine-induced Pancytopenia Indicating Bone Marrow Suppression: A Rare Pediatric Case Report from Pakistan

**DOI:** 10.7759/cureus.4462

**Published:** 2019-04-16

**Authors:** Shayan Marsia, Samar Mahmood, Mohammad Raza, Khushboo Nusrat, Ayesha Saleem

**Affiliations:** 1 Internal Medicine, Dow University of Health Sciences (DUHS), Karachi, PAK; 2 Pediatrics, Dow University of Health Sciences (DUHS), Karachi, PAK

**Keywords:** juvenile idiopathic arthritis, sulfasalazine, pancytopenia, bone marrow suppression, dmard, oligoarthritis, pediatrics, pakistan, joint pain, rheumatology

## Abstract

Juvenile idiopathic arthritis (JIA) is the most common chronic rheumatic condition in children. The treatment of JIA is mainly by drug therapy, which includes non-steroidal anti-inflammatory drugs (NSAIDs), corticosteroids, and disease-modifying antirheumatic drugs (DMARDs). Sulfasalazine is a DMARD that is used as the second-line of therapy. Although believed to have an effective and safe profile, it has side effects ranging from mild gastrointestinal discomfort to hematopoietic alterations. In this study, we present a case of JIA with sulfasalazine-induced bone marrow suppression in a five-year-old child, which is rarely reported within the pediatric age group across the literature.

## Introduction

Juvenile idiopathic arthritis (JIA) is the most common chronic rheumatic condition in children [1]. It is of unknown etiology; however, vital to the pathogenesis of the disease is the role of exogenous and endogenous antigens with an increased inflammatory response. The diagnosis is based on two factors: the onset of disease before 16 years of age and the presence of arthritis for more than six weeks [1]. In Europe and North America, the incidence of JIA ranges from two to 20 and from 16 to 150 per 100,000 children, respectively [1]. However, while considering different geographical and ethnic groups, there is a marked variation in the subtype of JIA, with oligoarthritis being more prevalent in the Western countries and polyarthritis predominating in Costa Rica, India, New Zealand, and South Africa [2-3]. In Asia, the majority of the cases of childhood arthritis present as systemic arthritis [3-4].

The criteria proposed by the International League of Associations for Rheumatology (ILAR) for systemic arthritis include arthritis with or preceded by quotidian fever lasting for a minimum of two weeks along with any one of the following: rash, generalized symmetrical lymphadenopathy, enlarged liver or spleen or serositis (pericarditis, pleural or pericardial effusion, and, rarely, peritonitis). In North America and Europe, 50%-80% cases of chronic arthritis in children present with oligoarthritis [2], in which four or lesser joints are involved during the first six months of the disease. The diagnosis is mainly clinical, with a detailed physical examination done on each visit, to assess all joints.

The treatment of JIA is mainly by drug therapy, which includes non-steroidal anti-inflammatory drugs (NSAIDs), corticosteroids, and disease-modifying antirheumatic drugs (DMARDs). Sulfasalazine is a DMARD that is used as the second-line of therapy. It is believed to have an effective and safe profile, as mentioned in a study by Rossum, et al. [5]. Its adverse effects range from mild gastrointestinal discomfort to hematopoietic alterations. In this study, we present a case of JIA with sulfasalazine-induced bone marrow suppression in a five-year-old child, which is rarely reported within the pediatric age group across the existent literature.

## Case presentation

A five-year-old male child presented to us in a tertiary-care, public hospital setting with fever, pallor, and rash over his body for five days. In addition, he suffered from two episodes of non-projectile, blood-stained vomitus and had developed a non-progressive, black lesion over his nose within the same period. As reported by the mother, his fever spiked around two months ago, was high grade, continuous, not associated with chills, and documented as going up to 103-104°F with an associated acute-onset earache and ear discharge. Four days after the onset of fever - swelling, pain, and limitation of movement were noted at the left ankle joint. The fever temporarily subsided by some medication prescribed at a local clinic and the joint pain was persistent, but the child was not further investigated at this point. Due to a lack of improvement of the symptoms, the mother had brought the child to the emergency room (ER) via which he was admitted to our pediatric ward and administered intravenous antibiotics over the course of the next two weeks, resulting in an improvement of symptoms (joint pain and fever). Following this, the relevant investigations were ordered and in view of the child’s symptoms not being completely alleviated by the antibiotics and his prolonged history, the case was discussed with a pediatric rheumatologist. The labs reported slightly elevated platelet count (451,000/ microliter), raised C reactive protein (CRP - 22.7mg/L), raised erythrocyte sedimentation rate (ESR - 42 mm/hr), and a negative antinuclear antibody test (ANA). The child was diagnosed as a case of oligoarticular juvenile idiopathic arthritis (JIA); treatment was started shortly after diagnosis and the patient was started on sulfasalazine (30 mg/kg/day, in two divided doses) and naproxen (15 mg/kg/day, in two divided doses). The parents were asked to seek an ophthalmologist’s opinion for his uveitis and advised regular follow-up.

After three weeks of treatment, the patient was brought back to the ER with the aforementioned complaints i.e. fever, pallor, rash, vomiting, and a black lesion on the nose for the past five days. On systematic review, the parents reported exertional dyspnea for the past five days. A system-wise conducted clinical examination revealed an increased heart rate (140 bpm), weak peripheral pulses, shifted apex beat with a notable gallop rhythm, bilateral basal crepts, palpable liver of 4 cm, multiple petechiae mainly over limbs and face, and a black colored ulcer at the tip of the nose, with pus discharge at the floor of the ulcer. Musculoskeletal and joint examination were unremarkable.

Based on the history and examination, the differential diagnoses included: JIA with macrophage activation syndrome (MAS), viral fever (dengue) and complicated malaria. Various investigations were carried out to reach a definitive diagnosis, including complete blood count, which showed low hemoglobin (6.3 g/dl), low MCV (71.4 fl), low platelets (2000/microliter), and severe neutropenia (180 cells/mm^3^). The malarial parasite was not seen and the dengue antigen was also negative. The urea, creatinine, and electrolytes came out normal. The other labs carried out revealed elevated CRP (223 mg/L) and ESR (42 mm/hr). In view of the suspicion for MAS; the liver function tests, fibrinogen, triglyceride, albumin, ferritin, and sodium levels were also checked, all of which were within their normal ranges. Sulfasalazine was stopped at this point due to the child’s extreme ill health.

Initially, the child was managed in the emergency department by administering oxygen, antibiotics (ceftazidime and amikacin), and paracetamol. He was later shifted to the pediatric intensive care unit (ICU) where methylprednisolone was started and platelets and packed RBCs were transfused. Further tests were performed, including blood peripheral smear preparation, which showed pancytopenia, and chest X-ray, which showed bilateral infiltrates.

The child was later shifted to the ward while there were still multiple spikes of high-grade fever. Pus was taken from the nose lesion and sent for culture. The differentials at this point included bacterial sepsis with immunosuppression and infective endocarditis. The culture returned positive for Pseudomonas aeruginosa and antibiotic therapy was accordingly changed to piperacillin/tazobactam, along with colomycin. With the change of antibiotic, the fever and lesion on the nose subsided. The child’s pediatric rheumatologist was consulted again regarding the case and sulfasalazine was restarted when he was stabilized, due to the strong suspicion of JIA. Upon resuming the drug, he first developed an allergic reaction on the same day, which was controlled with steroids and H1, H2 blockers, and he subsequently developed anemia and skin bleeding manifestations (and a generalized erythematous rash) on the successive days. The laboratory investigations run at this point portrayed pancytopenia (reticulocyte count 0.5%). At this time, it was strongly suspected that these manifestations were side effects of the prescribed sulfasalazine, so the drug was stopped and the patient managed conservatively. The child’s condition improved significantly with supportive management, after which he was discharged with instructions to follow-up at the outpatient department with a complete blood count (CBC) that remained within normal limits upon the two occasions it was reported at. Thereafter, the patient was advised regular follow-up.

In summary, the diagnosis was strongly believed to be that of sulfasalazine-induced bone marrow suppression, in view of how the patient had two episodes of pancytopenia (the first having been attributed falsely to MAS) and how his symptoms subsided shortly after stopping the drug.

## Discussion

In this case, we observed sulfasalazine-associated pancytopenia. Previous studies have shown that sulfasalazine-induced pancytopenia is caused by bone marrow suppression [6-7]. Although a bone marrow biopsy of the patient could not be performed due to the changing hematological profiles with the change in medicines, the pancytopenia in this patient, along with the onset of the symptoms of bone marrow suppression being immediately associated with the intake of sulfasalazine and alleviating shortly after the withdrawal of the drug on two occasions, were all factors indicative of bone marrow suppression induced by the drug itself.

In a systematic review conducted by Thierry et al. [8], more cases of JIA were reported in girls as compared to boys, indicating a gender predilection in the distribution of the disease. Although the literature does not provide sufficient evidence of sulfasalazine-induced bone marrow suppression in children, there is adequate proof available for it in teenagers greater than 16 years of age as well as in adults. Of remarkable importance is a lethal case of a 34-year-old woman who was being treated with sulfasalazine for seronegative rheumatic arthritis and who, on the 17th day of her treatment, developed a number of drug-induced adverse effects, including bone marrow suppression [9]. She passed away five weeks after the initiation of the drug therapy and her postmortem showed significant findings, such as hepatocellular necrosis, acute hypersensitivity myocarditis and focal acute tubulointerstitial nephritis. In order to correct the adverse effects, the timely withdrawal of the drug has a crucial role in the reversal of the symptoms and preservation of the quality of life. In a case involving a 22-year-old male patient being treated for ulcerative colitis and having developed bone marrow necrosis after four to five weeks of treatment, discontinuation of the drug resulted in complete recovery [10].

Bone marrow suppression in children is induced by a number of causes. It can be inherited (Fanconi anemia), viral (Epstein Barr virus), bacterial (Salmonella typhi), and parasitic (Plasmodium falciparum). However, the most common cause of acquired bone marrow suppression is aplastic anemia. Drug-induced bone marrow failure is encountered mostly with chemotherapeutic agents such as azathioprine. The diagnosis mostly depends upon biopsy; however, it was not performed in our patient. Another case similar to ours was diagnosed without a biopsy [11]; the hematologic profile being the source of support for the diagnosis.

Sulfasalazine is widely used for the treatment of rheumatoid arthritis/juvenile idiopathic arthritis. It has sulfapyridine and 5-aminosalicylic acid as its metabolites [12]. As illustrated by Smedegard and Bj˚ork (13), the effects of Sulfasalazine are dual. It not only serves as an immunomodulator by inhibiting the release of various cytokines, such as IL-1, IL-6, TNF-α, and others but also has anti-inflammatory properties, which act by reducing chemotaxis and superoxide production [13]. The bioavailability of sulfasalazine is 10%-30% after oral administration. The drug, which reaches the large intestine, is cleaved by bacterial enzymes called azoreductases into sulfapyridine and 5-aminosalicylic acid. More than 90% of sulfapyridine is absorbed from the large intestine whereas only 20%-30% 5-aminosalicylic acid is absorbed [14-15]. After systemic absorption, sulfasalazine is metabolized by the liver into sulfapyridine and 5-aminosalicylic acid [16] whereas a small amount is excreted in the urine unchanged [14-15]. Sulfapyridine undergoes acetylation and is excreted mainly in the urine [14-16], whereas the majority of 5-aminosalicylic acid is eliminated in feces [14-15].

Acetylation has a significant role in the development of adverse effects as the propensity increases with inherited acetylator phenotype and free serum sulfapyridine levels. Slow acetylators have higher levels of sulfapyridine and hence they manifest with adverse symptoms at equal doses as compared to the fast acetylators [17]. As 5-aminosalicylic acid does not undergo acetylation and it has been proven to have similar efficacy as sulfasalazine [18], it could be suggested as a viable alternative. However, in another study, 5-aminosalicylic acid has also been reported to cause pancytopenia [19].

Due to its effects on the hematopoietic system, it is recommended to urgently terminate the use of sulfasalazine upon encountering symptoms early in the treatment such as fever, chills, malaise, or other nonspecific symptoms that cannot be explained [20].

## Conclusions

Thus, we see a rare case of sulfasalazine-induced bone marrow suppression in a five-year-old child, with its detailed history and progression, associated investigations, differentials, and management. This case report highlights the need for pediatricians to keep bone marrow suppression as a dangerous side effect in mind when prescribing sulfasalazine to patients as well as for them to consider it as a cause of pancytopenia observed in children taking the drug. Lastly, this report urges other doctors in the field of pediatrics to report similar cases from within the age group so as to enable the depiction of a clearer picture regarding the condition in children.
